# Matrix Metalloproteinase 9 as a Predictor of Coronary Atherosclerotic Plaque Instability in Stable Coronary Heart Disease Patients with Elevated Lipoprotein(a) Levels

**DOI:** 10.3390/biom9040129

**Published:** 2019-03-29

**Authors:** Marat Ezhov, Maya Safarova, Olga Afanasieva, Maksim Mitroshkin, Yuri Matchin, Sergei Pokrovsky

**Affiliations:** Federal State Budget Institution “National Cardiology Research Center” of Ministry of Health of the Russian Federation; 15A, 3d Cherepkovskaya street, 121552 Moscow, Russia; dr.safarova@gmail.com (M.S.); afanasieva.cardio@yandex.ru (O.A.); mmg-doc@yandex.ru (M.M.); yumatchin@gmail.com (Y.M.); dr.pokrovsky@mail.ru (S.P.)

**Keywords:** matrix metalloproteinase 9, intravascular ultrasound, virtual histology, plaque, coronary disease, atherosclerosis, lipoprotein(a)

## Abstract

We sought to investigate whether levels of matrix metalloproteinases (MMPs) and their inhibitors predict coronary atherosclerotic plaque instability, as assessed by intravascular ultrasound (IVUS) virtual histology during coronary angiography. Blood samples were collected before angiography in 32 subjects (mean age 56 ± 8 years) with stable coronary heart disease (CHD) and elevated lipoprotein(a) (Lp(a), 94 ± 35 mg/dL). Levels of high-sensitivity C-reactive protein (hsCRP), apolipoprotein B100 (apoB100), MMP-7, MMP-9, tissue inhibitor of metalloproteinases (TIMP)-1, and TIMP-2 were determined using commercially available enzyme-linked immunosorbent assay kits. Results. The morphology of a total of sixty coronary lesions was assessed by virtual histology IVUS imaging. Eleven (18%) plaques in nine (28%) patients were classified as plaques with an unstable phenotype or a thin-cap fibroatheroma. Age, low-density lipoprotein cholesterol, apoB100, MMP-7, and MMP-9 levels were positively associated with necrotic core volume. Conversely, there was a negative relationship between MMP-7 and -9 levels and fibrous and fibro-fatty tissue volume. Multivariate regression analysis revealed that MMP-9 is a strong independent predictor of atherosclerotic plaque instability in stable CHD patients. In stable CHD patients with elevated Lp(a), MMP-9 levels are positively associated with the size of the necrotic core of coronary atherosclerotic plaques.

## 1. Introduction

Morphologic findings indicate that atheroma composition is not related to the degree of stenosis of the coronary artery, and about 40% of atherosclerotic plaques in patients with stable angina have a pool of extracellular lipids [1]. Precursors of plaque destabilization and evolution into a plaque at a high risk of rupture in the milieu of stable coronary artery disease (CAD) remain unclear. The phenotype of an unstable atheroma includes a thin fibrous cap, large lipid core, neovascularity, and aggregation of macrophages releasing inflammatory cytokines and proteases, including matrix metalloproteinases (MMPs). Plaques with a necrotic core >10% of their volume are highly susceptible to disruption, causing an ischemic event [2,3,4,5]. The local increase in MMP secretion contributes to plaque destabilization through augmentation of collagen and elastin destruction and fibronectin cleavage. A simultaneous counterbalancing increase in the local expression of anti-inflammatory signaling pathways promotes extracellular matrix synthesis, hence preventing remodeling and degradation within the plaque. For instance, tissue inhibitors of metalloproteinases (TIMPs) reduce MMP activity [6].

Most clinical studies have evaluated MMP levels and plaque structure and morphology in patients with acute coronary syndrome (ACS) [7,8] or have compared patients with unstable and stable angina [9,10]. Previously, we conducted a prospective controlled trial, Specific Lp(a) apheresis for regression of coronary and carotid atherosclerosis (LaRCA), which investigated the effect of specific lipoprotein(a) (Lp(a)) apheresis on angiographic coronary disease in patients with stable coronary heart disease (CHD) and elevated Lp(a) levels (NCT02133807; clinicaltrails.gov). The purpose of the present post-hoc analysis was to evaluate the levels of selected MMPs and TIMPs and their association with coronary plaque morphology based on the virtual histology intravascular ultrasound (VH IVUS) data prior to initiation of treatment with apheresis.

## 2. Materials and Methods

The LaRCA design was described elsewhere [11]. Briefly, all patients with stable CHD and Lp(a) ≥50 mg/dL who had clinical indications for left heart catheterization provided written informed consent for participation. The study was approved by the Myasnikov Institute of Clinical Cardiology Review Board (Moscow, Russia). Subjects underwent IVUS during coronary angiography. Eligibility criteria for the target coronary lesion included the following: 20–60% narrowing of the luminal diameter in a vessel with a diameter >2 mm; no previous intervention in the vessel; extent of calcification ≤2; absence of marked tortuosity or occlusion in the distal segment of the studied vessel or stenosis >70% in the secondary branch. Patients were excluded if they had ACS within 30 days prior to screening; familial hypercholesterolemia; triglyceride (TG) levels >4.5 mmol/L (400 mg/dL); uncontrolled diabetes, uncontrolled hypertension or severe heart failure; renal or thyroid dysfunction; significant liver disease; or treatment with other than statins as lipid-lowering medications. Individuals requiring urgent myocardial revascularization were excluded.

Medical history, physical examination, and the results of the stress test were ascertained prior to coronary angiography. The coronary angiographic assessment was performed using the Allura Xper FD10 system (Philips, Best, The Netherlands) via radial or ulnar access with the 6F introducer. The image of the left coronary artery was registered in standard orthogonal projections, and the image of the right coronary artery was registered in two standard projections. The contrast agent (7–10 mL) was injected manually at a rate of 2–3 mL/s; imaging speed was 15 frames/s. The automatic quantitative coronary analysis (QCA) of the angiograms was carried out using the Xcelera digital system (Philips). To perform QCA, we used the final diastolic frame of the projection to visualize the stenosis maximum. The tip of the guiding catheter, which was not filled with the contrast agent, was used for calibration. Six patients had single-vessel disease, while two and three affected major coronary arteries were in 10 and 16 patients, respectively. Major coronary arteries or large secondary branches that met the eligibility criteria were selected for IVUS examination. Following coaxial positioning of the guiding catheter in the ostium of the left or right coronary artery, the intracoronary transducer was passed into the distal segment. The ultrasound catheter was guided along the transducer distally to the region of interest of the coronary artery. Before the IVUS procedure was initiated, all patients underwent intracoronary administration of 250 μg of nitroglycerin. The IVUS data was registered using continuous back traction with a mechanical pull-back device at 0.5 mm/s with the electronic ultrasound sector scanner Eagle Eye Gold^™^, which had an operating frequency of 20 MHz (Volcano Therapeutics, Inc., Rancho Cordova, CA, USA). The traction was performed up to the tip of the guiding catheter. While the transducer was moving, the data were transferred to the Volcano S5 console (Volcano Therapeutics, Inc.) where they were synchronized with the electrocardiogram (ECG). The radiofrequency analysis of the ultrasound signal (VH) and imaging was synchronized with the R wave on ECG. The atherosclerotic plaque margins were defined based on the coronary angiography data as the most unchanged sections/areas of an artery in the proximal and distal directions from the site of interest. Following the procedure, the plaque was assessed along its full length, with manual adjustments of intima-lumen and media-adventitia boundaries for each imaging frame of the selected segment.

The investigated vessels were distributed as follows: left main stem—1, left anterior descending artery—15, circumflex—24, and right coronary artery—20. Four plaque components were identified based on the VH IVUS data, namely, the fibrous, fibro-lipid, necrotic, and calcification components [12]. The following parameters were measured in the automatic mode with manual correction: total atheroma volume (TAV in mm^3^), volumes of all four plaque components (in mm^3^), and percentages of TAV. The plaques were classified into four types: pathological intimal thickening (PIT), fibroatheroma, calcified fibroatheroma, and thin cap fibroatheroma (TCFA) [3].

Blood samples were drawn immediately prior to angiography and stored at −20 °C or −70 °C in accordance with the directions of the kit manufacturer. The concentrations of total cholesterol (TC), TG, high-density lipoprotein cholesterol (HDL-C), apolipoprotein B100 (apoB100), and high-sensitivity C-reactive protein (hsCRP) were determined enzymatically using the Architect 8000 analyzer (Abbott, Abbott Park, IL, USA). The Friedewald equation was used to calculate low-density lipoprotein cholesterol (LDL-C). An enzyme-linked immunoassay (ELISA) from R&D Systems (Minneapolis, MN, USA) was used to measure the MMP-7, MMP-9, and TIMP-2 concentrations in serum, and an ELISA from Bender Medsystems GMBH (Vienna, Austria) was used to determine TIMP-1 levels. The hsCRP level was measured using an ELISA from Cytimmune Sciences, Inc. (Rockville, MD, USA).

Values with normal distribution are presented as the mean ± standard deviation (SD), while those with abnormal distribution are expressed as the median (25–75% quartiles). Spearman’s correlation analysis was performed to evaluate the association between biomarkers and plaque components. All variables that showed significant correlation in univariate analysis were included in the multiple regression model to determine the most powerful predictors of necrotic plaque volume. Two-tailed *P*-value < 0.05 was considered statistically significant. Statistica^®^ software (version 10.0, StatSoft, Inc., Tulsa, OK, USA) was used to perform the analyses.

## 3. Results

There were 32 eligible patients (age range 37–70 years) who were enrolled in this study (Table 1). About two-thirds (63%) of the included patients were men, and half of the cohort was diagnosed with hypertension. The mean time since the diagnosis of CHD was 5.5 years; more than half of the patients had a history of myocardial infarction (MI) and/or undergone coronary revascularization. All patients had been taking low-dose aspirin and a statin for a mean period of 3.4 years prior to enrollment.

**Figure 1:**
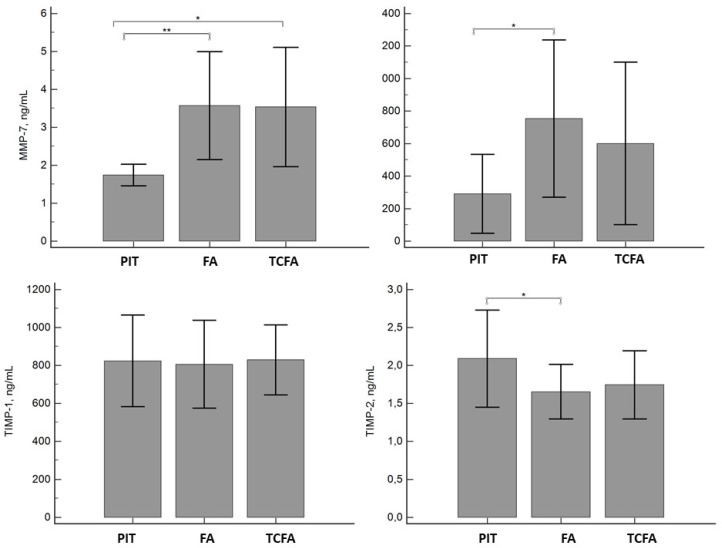
Distribution of the levels of matrix metalloproteinase (MMP)-7 and -9 and tissue inhibitors of matrix metalloproteinase (TIMP)-1 and -2 depending on the coronary plaque phenotype. The data are presented as the mean ± standard deviation. * *P* <0.05; ** *P* <0.005, as compared to PIT. PIT, pathological intimal thickening; FA, fibroatheroma; TCFA, thin cap fibroatheroma.

Sample size calculations were based on the IVUS evaluations, which showed 60 plaques in 38 target arteries. Grey-scale IVUS demonstrated a mean TAV of 136 ± 91 mm^3^ in this cohort. Eleven (18%) plaques in nine (28%) subjects were classified as TCFA. Also, 42 fibroatheromas, 6 PITs, and 1 calcified fibroatheroma were identified. The presence of a fibroatheroma phenotype was associated with higher levels of MMP-7 and MMP-9 and lower levels of TIMP-2 when compared to PIT (Figure 1). The unstable plaque phenotype, TCFA, was associated with high MMP-7 levels.

Table 2 shows univariate association analyses between necrotic core size and age, levels of LDL-C, apoB100, MMP-7, and MMP-9. There was an inverse relationship between MMP-7 and MMP-9 levels and the volume of the fibrous component of the plaque; levels of TIMP-1 and MMP-9 were negatively correlated with the relative amount of fibrous-fatty tissue. In the multivariate regression analyses adjusted for age, sex, and including apoB100, MMP-7 and -9, only the level of MMP-9 was independently predictive of the size of the necrotic core (β = 0.44, *P* < 0.05). Expectedly, there was a negative relationship between MMP-9 and TIMP-1 (r = −0.28, *P* = 0.044) and a positive association between TIMP-1 and TIMP-2 (r = 0.40, *P* = 0.003). Of note, there was no significant association between the levels of hsCRP or Lp(a) with any of the plaque components.

## 4. Discussion

This study provides evidence that elevated levels of MMP-7, MMP-9, and apoB100 are directly associated with the size of the necrotic core of the coronary atherosclerotic plaque, as assessed by VHIVUS in patients with stable CHD and elevated Lp(a). Further, MMPs and not apoB100 were negatively correlated with the relative amount of plaque fibrosis. Fibro-lipid component of the plaque was directly related to the level of TIMP-1. MMP-9 has been found to be an independent predictor of plaque instability.

Given the intrinsic limitations of the study design, the causality of this relationship with necrotic tissue in the plaque could not be established. It has been shown that MMP expression is predominantly increased in the shoulder region of the atherosclerotic plaque [13]. The ability of MMPs to destroy connective tissue in the fibrous cap leads to a relative increase in the size of the necrotic core [6]. The resultant degradation of the collagen tissue in the plaque cap increases the risk of plaque destabilization and rupture [6]. The synthesis of MMPs is known to be driven by subendothelial macrophages. However, there is a lack of data pertinent to the inciting event promoting the sudden release of MMPs in patients with stable CAD with well-controlled conventional cardiovascular risk factors and who are compliant with their guideline-directed medical therapy.

Previously, it has been shown that individuals presenting with ACS and unstable plaques have two to three times higher levels of MMP-9 compared to subjects with stable CAD [7]. The expression of MMP-9 is elevated in debris from distal protective vascular guards in patients with ACS but not with stable angina [8]. In a cross-sectional study of 188 patients, the level of MMP-9 was significantly higher in patients with ACS (*n* = 82) and significantly associated with the presence of ruptured plaques. There was a weak positive correlation between the absolute area of the necrotic core and MMP-9 levels (r = 0.252, *P* = 0.002). Notably, other parameters, including hsCRP, MMP-2, TIMP-1, and lipids, were comparable in subjects with and without ruptured plaques [9]. In our study focusing on patients with elevated Lp(a), this relationship between MMP-9 levels and necrotic core size was more accentuated. Further, we also found that the levels of MMP-7 had a consistent relationship with characteristic features of plaque instability.

In contrast, Ko et al. evaluated 70 patients with stable and unstable angina and found no differences in the serum levels of hsCRP, MMP-9, or neopterin between the groups [10]. No relationship between these biomarkers and the structural characteristics of coronary plaque was reported except for a weak association between the neopterin level and the absolute volume of the necrotic core (r = 0.320, *P* = 0.008). It is worth noting that despite the younger age of our patient population (mean age 56 years vs. 61 years in [10]), the mean necrotic core volume was 1.5 times greater (23% vs. 15% in [10]). In another two studies in patients with ACS, the age group was close to the cohort of Ko et al., while the plaque characteristics acquired with VH IVUSwere comparable to those in the present study (necrotic core volume 32% and 25% vs. 23% in the present study) [9,13]. We speculate that in order to detect a relationship between biomarkers and plaque components, the mean necrotic core volume should be at least 10%. It has been shown that elevated serum levels of MMP-9 but not levels of MMP-1, MMP-3, or monocyte chemoattractant protein-1 were independently associated with carotid artery plaque instability [14,15]. In a large prospective trial that included 1127 patients with documented CAD, the association between MMP-9 levels and the risk of fatal cardiovascular events showed a hazard ratio of 1.3 (95% CI (confidence interval), 1.1 to 1.6; *P* = 0.005) after adjustment for clinical and therapeutic confounders [16]. We suggest that high levels of MMP-9 can be utilized as a marker of coronary plaque vulnerability. Further studies are needed to investigate the translation of these findings into hard-outcome data, specifically cardiovascular mortality, MI, stroke.

There is a paucity of data evaluating the association of the levels of MMP-7 with clinical manifestations of atherosclerosis. To our knowledge, these data provide the first outlook on the relationship between the levels of MMP-7 and features of plaque instability in patients with stable CAD and high Lp(a) levels. Matrix metalloproteinase 7 is related to vascular smooth muscle cell apoptosis and is associated with the lipid-laden macrophages along with the necrotic core of the lesions, supporting its important role in plaque destabilization [17]. Matrix metalloproteinase 7 is produced by activated macrophages and has a potent capacity to degrade numerous matrix components, including proteoglycans, insoluble elastin, and fibronectin [18]. Abbas et al. found that MMP-7 levels were significantly higher in 182 patients with asymptomatic and symptomatic carotid stenosis (median 1.96 ng/mL) than in 23 controls (median 0.83 ng/mL; *P* < 0.001) [19]. Immunohistochemical analysis of carotid plaques showed that MMP-7 was located primarily in macrophages and was found predominantly in areas with less organized collagen fibers. In the multivariate model, only age and MMP-7 (HR 1.4, *P* < 0.001) were identified as significant predictors of all-cause mortality during the mean follow-up of 3.5 years. The plasma MMP-7 concentration was unrelated to other inflammatory markers, suggesting another mechanism for MMP-7 involvement in atherogenesis [20]. In our study, the levels of MMP-7 were directly associated with the necrotic core size of the coronary atheroma.

We found that MMP-9 levels were negatively correlated with TIMP-1 (r = −0.28, *P* = 0.044), exhibiting MMP-inhibiting potential [21,22]. In turn, TIMP-1 was negatively associated with the relative volume of fibrous-fatty tissue. Experimental studies have demonstrated that TIMP-1 levels are unchanged or even elevated in atherosclerotic plaques [21,22]. In the prospective AtheroGene study (*n* = 1979), levels of TIMP-1 (HR 1.30, 95% CI, 1.07–1.58), hsCRP (HR 1.79, 95% CI, 1.43–2.24), and B-type natriuretic peptide (HR 2.75, 95% CI, 1.94–3.89) were independently associated with cardiovascular death in CAD patients [23]. It is important to note that the mean concentration of TIMP-1 was higher in patients who experienced a fatal cardiovascular event and had three-vessel disease than those who did not. The TIMP-1 concentration was significantly increased in patients with unstable coronary disease and showed a positive correlation with the number of leukocytes, particularly neutrophilic granulocytes, in CHD patients [20].

There was a positive relationship between TIMP-2 and TIMP-1, signifying a unidirectional effect. In addition to having an inhibitory effect on MMPs, TIMP-2 has the ability to directly suppress the proliferation of endothelial cells [24]. For the first time, our study demonstrated that the TIMP-2 level showed correspondence with plaque morphology, although no significant associations were found.

Here, we demonstrated that apoB100, but not hsCRP, is linked to necrotic core volume. Kubo et al. used VH IVUSto evaluate the relationship between serum hsCRP levels and coronary plaque composition in patients with stable angina. They found that the necrotic core in the plaques of patients with hsCRP levels >3 mg/L were significantly larger than in patients with normal hsCRP levels (20 ± 9% vs. 16 ± 8%, *P* = 0.014) [25]. The relative size of the necrotic core was positively correlated with the serum level of hsCRP (r = 0.20, *P* = 0.037). The absence of any relationship between hsCRP, MMPs and plaque components in our study could be explained by the prolonged and regular statin therapy. As was shown previously, statins dramatically inhibit the secretion of MMP-9 from macrophages and smooth muscle cells [26]; however, it appears that statin therapy does not affect the association between MMP-9 levels and plaque components.

To the best of our knowledge, this is the first study to demonstrate an association between apoB100 levels and the IVUS-determined relative size of the necrotic core. The association between apoB100-containing lipoproteins and four tissue plaque components included in the VH IVUS algorithm was evaluated previously in only one single-center study that included 60 patients with clinical indications of coronary angiography [13]. This known association of apoB-containing lipoproteins with larger plaque burden and a higher proportion of non-calcified plaques is in agreement with our results, regardless of the chronic statin therapy in both studies. In another study of 50 patients with ACS and borderline culprit lesions, stepwise multivariate linear regression analyses showed that changes in MMP-9 and hsCRP levels, but not TIMP-1 and apoB100 levels, in response to 12 months of atorvastatin treatment predicted the stabilization of vulnerable plaques [27].

The role of Lp(a) as an independent cardiovascular risk factor involved in atherogenesis, thrombosis, and inflammation has been actively studied and discussed [28]. In stable CHD patients, an elevated Lp(a) level (>50 mg/dL) was associated with nonclassical or intermediate monocytes (CD14++ CD16+) [29], which in turn produce MMP-9 [30], was related to TCFA [31], and independently predicted cardiovascular events in subjects referred for elective coronary angiography [32].

Study limitations. The small sample size of the present study was compensated by the evaluation of 60 coronary plaques. A controlled setting with the implementation of chronic medical therapy may have led to an underestimation of some variables in the final analysis. We did not analyze all known MMPs and their inhibitors, but of those that were measured, we demonstrated that MMP-9 was the most important factor for plaque vulnerability in patients with stable CHD. All enrolled patients had Lp(a) levels >50 mg/dL, which could have skewed this population toward a higher risk than a standard cohort of patients with stable ischemic heart disease, allowing for a more prominent effect size. Unrecognized biases might have led to the selection of patients fulfilling IVUS criteria referred for Lp(a) examination. However, the inclusion of consecutive patients minimized selection bias.

## 5. Conclusions

Serum apoB100, MMP-7, and MMP-9 levels in stable CHD patients were positively associated with the size of the necrotic core of atherosclerotic plaques and were inversely related to the fibrous tissue content of the plaques. MMP-9 is a promising marker of coronary plaque instability in patients with elevated Lp(a) levels.

## Figures and Tables

**Table 1:** Characteristics of 32 study participants.

Parameter	Value
Age, years	56.1 ± 8.0
Males	20 (62.5%)
Arterial hypertension	17 (53%)
Smoking	15 (47%)
Type 2 diabetes	2 (6%)
Family history of CHD	16 (50%)
Body mass index, kg/sq. m	27.1 ± 2.3
Angina pectoris, III–IV class	16 (50%)
Myocardial infarction	17 (53%)
Coronary artery bypass grafting	2 (6%)
Percutaneous coronary intervention	17 (53%)
CHD duration, years	5.5 ± 6.4
Statin intake, years	3.4 ± 2.1
**Biomarkers**	
TC, mmol/L (mg/dL)	4.8 ± 1.1 (186 ± 43)
LDL cholesterol mmol/L (mg/dL)	2.7 ± 0.8 (104 ± 31)
HDL cholesterol, mmol/L (mg/dL)	1.3 ± 0.4 (50 ± 16)
TG, mmol/L (mg/dL)	1.5 ± 0.6 (133 ± 53)
Lipoprotein(a), mg/dL	94 ± 35
ApoB100, mg/dL	92 ± 23
hsCRP, mg/L	1.3 (0.7–3.0)
MMP-7, ng/mL	3.4 ± 1.5
MMP-9, ng/mL	679 ± 483
TIMP-1, ng/mL	812 ± 221
TIMP-2, ng/mL	1.7 ± 0.4
**Intravascular ultrasound characteristics**	
Total atheroma volume, mm³	136 ± 91
Necrotic core, mm³	22.0 ± 21.7
Dense calcium, mm³	9.0 ± 13.3
Fibrous tissue, mm³	50.9 ± 36.4
Fibro-fatty tissue, mm³	9.8 ± 11.9
Necrotic core, %	23 ± 10
Dense calcium, %	9 ± 7
Fibrous tissue, %	57 ± 11
Fibro- fatty tissue, %	11 ± 7
**Baseline treatment**	
Acetylsalicylic acid	30 (94%)
Clopidogrel	13 (37.5%)
Angiotensin-converting enzyme inhibitor	18 (56%)
Angiotensin receptor antagonists	7 (26%)
Beta blockers	27 (84%)
Calcium channel blockers	11 (34%)
Nitrates	13 (41%)
Atorvastatin	32 (100%)

The data are presented as the mean ± standard deviation; median (25%–75%) or n (%); CHD, coronary heart disease, TC, total cholesterol; TG, triglycerides; LDL, low-density lipoprotein; HDL, high-density lipoprotein; ApoB100, apolipoprotein B-100; hsCRP, high sensitivity C-reactive protein; MMP, matrix metalloproteinase; TIMP, tissue inhibitor of metalloproteinase. For total, HDL, and LDL cholesterol multiply mmol/L by 38.67; for triglycerides multiply mmol/L by 88.57.

**Table 2:** Association between atherosclerotic risk factors, lipids and lipoproteins, biomarkers and plaque components, as assessed by intravascular ultrasound and virtual histology.

	TAV, mm^3^	NC, %	DC, %	FT, %	FF, %
Age	0.16	0.46	0.46	−0.54	−0.28
	0.10	0.013	0.011	0.003	0.14
Hypertension	0.10	0.30	0.27	−0.27	−0.31
	0.9	0.11	0.15	0.16	0.1
Smoking	−0.17	−0.14	−0.08	0.05	0.26
	0.36	0.47	0.67	0.79	0.18
CHD family history	−0.06	0.08	0.06	−0.04	−0.15
	0.75	0.67	0.77	0.85	0.43
LDL-C	−0.19	0.36	−0.07	−0.18	−0.14
	0.32	0.06	0.73	0.35	0.46
HDL-C	−0.16	0.02	−0.16	0.14	−0.13
	0.4	0.91	0.42	0.47	0.49
TG	-0.04	0.14	0.09	−0.12	−0.11
	0.82	0.47	0.63	0.52	0.57
ApoB100	−0.21	0.44	0.07	−0.29	−0.24
	0.27	0.018	0.70	0.13	0.21
Lp(a)	0.33	0.30	0.24	−0.29	−0.21
	0.06	0.1	0.19	0.11	0.24
hsCRP	−0.07	0.36	0.18	−0.29	−0.23
	0.72	0.06	0.35	0.13	0.23
MMP-7	−0.05	0.60	0.31	−0.53	−0.35
	0.82	0.001	0.1	0.003	0.06
MMP-9	0.26	0.49	0.26	−0.37	−0.39
	0.18	0.007	0.18	0.047	0.037
TIMP-1	0.05	0.18	0.21	−0.08	−0.4
	0.8	0.36	0.28	0.67	0.031
TIMP-2	0.04	−0.15	−0.18	0.2	0.06
	0.83	0.45	0.35	0.29	0.74

For each tested variable, the upper row contains r (the correlation coefficient) and the lower row represents the *P*-value (the level of significance). TAV, total atheroma volume; NC, necrotic core; DC, dense calcium; FT, fibrous tissue; FF, fibro-fatty; CHD, coronary heart disease; TG, triglycerides; LDL-C, low-density lipoprotein cholesterol; HDL_C, high-density lipoprotein cholesterol; ApoB100, apolipoprotein B-100; hsCRP, Lp(a), lipoprotein(a); high sensitivity C-reactive protein; MMP, matrix metalloproteinase; TIMP, tissue inhibitor of metalloproteinase.
